# Milk

**Published:** 1860-10

**Authors:** 


### MILK.

It was a sight which brought with it pleasant memories when standing at the Spring Garden entrance of St. James's Park, London, within a few rods of the palace of her gracious Majesty Victoria the First—long live the same!—we have contemplated a multitude of nurses with children of every age, size, and sex, gathered around a magnificent cow, the cup of each child sent up in its turn to catch the luscious fluid as it flowed all fresh and sweet and pure and rich from its natural fountain, to be transferred in a trice to expectant lips, which would fairly smack with delight and in another instant ask for "more!" This is the method which cousin John Bull, sturdy and practical as he is, adopts to secure to his little calves the real, original, identical juice of the—cow. We do not by any means pretend to emulate him in all things, although in some we do excell; in brag and fight, for example! But New-Yorkers can do better. On a great emergency, warm milk is passable, say when you have not had any thing to eat or drink for a week; but to have it from a sparkling, clear goblet, creamy, pure, and cool as the water which drops from the

"Moss-covered bucket that hangs in the well,"

is certainly worth going a little out of the way for. Such a pleasure we will guarantee any dear lover of milk, who will choose to call at 246 Tenth street, a very few doors east of Broadway, where it is delivered from real farm-house cows, having been drawn a few hours before, and brought in by the Erie cars, in double-quick time. We make a proposition to the sweetest "seventeen" within a hundred miles of Gotham, to get up a fairy little office on Broadway, and vend this milk in penny glasses for children, and half-dimes for older people. What a delicious draught it would be for invalids and feeble persons, for children going to or returning from school, and how infinitely preferable to soda-water and root-beer, to gin sling, brandy toddy, sangaree or lager. Is there no enterprising Barnum to work up this suggestion into practical operation, to bless all Gothamite juvenility, and give health and nutriment and vigor to the sad sons and daughters of sickness and suffering? Why might it not—why should it not succeed?

